# A two-decade comparison of prevalence of dementia in individuals aged 65 years and older from three geographical areas of England: results of the Cognitive Function and Ageing Study I and II

**DOI:** 10.1016/S0140-6736(13)61570-6

**Published:** 2013-10-26

**Authors:** Fiona E Matthews, Antony Arthur, Linda E Barnes, John Bond, Carol Jagger, Louise Robinson, Carol Brayne, on behalf of the Medical Research Council Cognitive Function and Ageing Collaboration

**Affiliations:** a MRC Biostatistics Unit, Cambridge Institute of Public Health, Cambridge University, Cambridge, UK; b School of Nursing Sciences, University of East Anglia, Norwich, UK; c Department of Public Health and Primary Care, Cambridge Institute of Public Health, Cambridge, UK; d Institute of Health and Society, Faculty of Medicine, Newcastle University, Newcastle, UK

## Abstract

**Background:**

The prevalence of dementia is of interest worldwide. Contemporary estimates are needed to plan for future care provision, but much evidence is decades old. We aimed to investigate whether the prevalence of dementia had changed in the past two decades by repeating the same approach and diagnostic methods as used in the Medical Research Council Cognitive Function and Ageing Study (MRC CFAS) in three of the original study areas in England.

**Methods Between:**

1989 and 1994, MRC CFAS investigators did baseline interviews in populations aged 65 years and older in six geographically defined areas in England and Wales. A two stage process, with screening followed by diagnostic assessment, was used to obtain data for algorithmic diagnoses (geriatric mental state–automated geriatric examination for computer assisted taxonomy), which were then used to estimate dementia prevalence. Data from three of these areas—Cambridgeshire, Newcastle, and Nottingham—were selected for CFAS I. Between 2008 and 2011, new fieldwork was done in the same three areas for the CFAS II study. For both CFAS I and II, each area needed to include 2500 individuals aged 65 years and older to provide power for geographical and generational comparison. Sampling was stratified according to age group (65–74 years *vs* ≥75 years). CFAS II used identical sampling, approach, and diagnostic methods to CFAS I, except that screening and assessement were combined into one stage. Prevalence estimates were calculated using inverse probability weighting methods to adjust for sampling design and non-response. Full likelihood Bayesian models were used to investigate informative non-response.

**Findings:**

7635 people aged 65 years or older were interviewed in CFAS I (9602 approached, 80% response) in Cambridgeshire, Newcastle, and Nottingham, with 1457 being diagnostically assessed. In the same geographical areas, the CFAS II investigators interviewed 7796 individuals (14 242 approached, 242 with limited frailty information, 56% response). Using CFAS I age and sex specific estimates of prevalence in individuals aged 65 years or older, standardised to the 2011 population, 8·3% (884 000) of this population would be expected to have dementia in 2011. However, CFAS II shows that the prevalence is lower (6·5%; 670 000), a decrease of 1·8% (odds ratio for CFAS II *vs* CFAS I 0·7, 95% CI 0·6–0·9, p=0·003). Sensitivity analyses suggest that these estimates are robust to the change in response.

**Interpretation:**

This study provides further evidence that a cohort effect exists in dementia prevalence. Later-born populations have a lower risk of prevalent dementia than those born earlier in the past century.

**Funding:**

UK Medical Research Council.

## Introduction

Dementia is a topic of international concern.^1^ Governments worldwide want to commit to action, including primary prevention (reduction of risk factors), secondary prevention (detection of proven prodromal and preclinical states, during which early intervention has proved better than usual presentation), and tertiary prevention (best possible care for individuals with manifest dementia). This attention has been driven by epidemiological evidence, for existing populations and projected into the future,^2^ combined with increased societal concern.^3^ Generation of such epidemiological evidence is challenging. Population-based prevalence studies involve the identification or creation of true population sampling frames—including individuals living in care homes—and recruitment of communities and individuals to take part in the detailed assessments that are needed for a robust diagnosis. Prevalence studies detect unrecognised cases and those previously recognised by the family or health-care and social-care services.

There has been an increase in epidemiological dementia research since the 1980s, with attempts at harmonisation across geographical areas, using common methods and diagnostic criteria. The estimates from these studies have been the basis of Delphi and other reviews.^1^, ^2^, ^4^, ^5^ These estimates have been used to update projections of present and future demand for dementia-care services.^1^ Carers and charitable organisations have exerted pressure on policy makers, resulting in increased investment in studies of prevalence and incidence in countries of lower and middle income, and development of dementia-specific services and research policy in higher income countries. However, despite an emphasis on investment in research investigating the detailed biological underpinnings of neurodegenerative disorders, few new studies have examined potential changes in the prevalence of dementia across time in specific age groups.^6^, ^7^, ^8^ Governments and policy makers need accurate contemporaneous data, but the estimates for the UK and those widely used for estimation in higher income countries are now 20 years old.^9^, ^10^

The prevalence of dementia in the population might be subject to change. Factors that might increase prevalence include: rising prevalence of risk factors, such as physical inactivity, obesity, and diabetes; increasing numbers of individuals living beyond 80 years with a shift in distribution of age at death;^11^ persistent inequalities in health across the lifecourse;^12^ and increased survival after stroke and with heart disease. By contrast, factors that might decrease prevalence include successful primary prevention of heart disease, accounting for half the substantial decrease in vascular mortality,^13^ and increased early life education, which is associated with reduced risk of dementia.^14^

Few studies can examine change over time. Studies based on routine data obtained over decades in the USA have hinted at reductions in dementia and severe cognitive impairment,^15^, ^16^ as have single-site studies in Europe;^6^, ^7^, ^8^, ^17^ however, there is some conflicting evidence from Asia.^18^ This optimism has been tempered because of the widely differing methods of these studies: from population identification and health-care access to potentially subtle changes in diagnostic and sampling methods and limitations of analytical approaches.

The first UK Cognitive Function and Ageing Study (CFAS), known as the Medical Research Council (MRC) CFAS, began in 1989. One of a suite of European prevalence and incidence studies (forming the EURODEM collaboration),^19^ it was designed to test for geographical differences within the UK, across populations with widely varying characteristics, including vascular health. No consistent differences were identified across Europe or within the UK.^19^ MRC CFAS examined populations in six geographical areas, with samples taken from the entire resident population, including those in care homes, and common diagnostic methods (five areas had identical methods). The response was about 80%. Prevalence and incidence estimates from MRC CFAS have been used extensively for national and international purposes.^5^, ^9^, ^20^

Since these European studies were initiated there has been much work in genetics, biology, and imaging. New investigations and classifications have been introduced, including the widespread uptake of clinical criteria for an intermediate stage of cognition with increased risk of dementia progression (mild cognitive impairment), creating an unstable diagnostic environment. In most epidemiological studies since the late 1990s, starting in the USA, a consensus multidisciplinary diagnostic process has been used to agree diagnostic criteria, themselves in a state of flux.^21^ It is highly likely that changes in diagnostic criteria and the increased recognition of mild cognitive impairment as a new early preclinical diagnosis has led to shifting boundaries, such that it might be difficult to compare old estimates with new ones. CFAS has the potential to allow such a comparison over time.

A new generation study, CFAS II, was designed and funded to test whether the prevalence of dementia (and incidence of dementia in due course) had changed from 1991 to 2011.

## Methods

### Setting, study design, and participants

Between 1989 and 1994, the MRC CFAS investigators did baseline interviews in populations aged 65 years and older in six geographical areas in England and Wales. A two stage process, with screening followed by diagnostic assessment, was used to estimate dementia prevalence. Data from three of the English areas of MRC CFAS—Cambridgeshire, Newcastle, and Nottingham, where interviews were done between Dec 19, 1990, and July 6, 1993—were selected to provide CFAS I estimates. Between Nov 13, 2008, and Oct 25, 2011, we did new fieldwork in the same geographical areas to provide CFAS II estimates, which could be directly compared with CFAS I. These areas were selected to provide a geographical spread, including a rural area.

CFAS I and CFAS II had identical designs and methods, apart from a change from a two-stage design with CFAS I to a one-stage design with CFAS II. In CFAS I, the first phase was a screening process, which identified a subset of individuals who underwent diagnostic assessment. CFAS II combined the screening and assessment phases. More details are provided in the protocol.

Both CFAS I and CFAS II drew on the UK system of primary care registration, which provides the most robust population base for sampling by age group for epidemiological studies in the UK. In both studies, the population for invitation to interview was randomly sampled from the same geographical areas. Each area needed to include 2500 individuals aged 65 years and older to provide power for geographical and generational comparison. Sampling was stratified according to age group (65–74 years *vs* ≥75 years; 1250 per stratum per area). We used oversampling to allow for losses (death, incorrect registration, ineligibility, general practitioner refusals, participant or gatekeeper refusal). The primary care practices screened records of patients in selected samples regularly for deaths and terminal illness. An introductory letter from the general practitioner was followed by a visit by a named study interviewer. Fully informed written consent was sought, and when capacity was impaired procedures complied with the UK Mental Capacity Act 2005.

### Procedures

For both CFAS I and CFAS II, we recruited local interviewers from a range of backgrounds (those working with older people in the voluntary, health-care, and social-care sectors), and gave them identical training (overseen by one investigator) to deliver the standardised computerised interviews. This training consisted of an intense week-long course, with follow up practice until each interviewer achieved the necessary quality standards, with ongoing quality control. Interviewers visited residences up to three times, and whenever possible elicited a response from the intended respondent. Individuals who had moved but remained within the geographical area were included for interview.

The CFAS I baseline interview included questions about sociodemographic characteristics, lifestyle, health, activities of daily living (basic and instrumental), cognition, health-care and social-care contact, and medication. A sample of 20% of those who had a baseline interview, stratified to represent the entire cognitive spectrum, was invited for assessment with the geriatric mental state (GMS) examination, a standardised interview for ascertainment of dementia and other neuropsychiatric syndromes in the older population. This examination provided the data for the diagnostic algorithm, the automated geriatric examination for computer assisted taxonomy (AGECAT; drawing on respondent and observer ratings).^22^ For all these respondents we also requested an informant interview, which included more detail on the presence of symptoms and sympton duration. In CFAS II, a one-stage interview was done, which integrated the screening and assessment phases of MRC CFAS to reduce analytical complexity and attrition. An informant interview for those with a cognitively impaired profile, and for a random selection of those without, was requested for 20% of the sample. The content of the interviews is provided on the CFAS website.

CFAS I and CFAS II used an algorithmic approach to diagnosis to provide consistency across area and time, eliminating the variability that has been shown with clinical diagnosis.^23^ The principle of the process is that data needed for diagnosis are collected in a standardised manner, and a diagnostic algorithm is then applied to these data. GMS-AGECAT diagnoses have been shown to be as reliable as those made by a range of clinicians and has been validated against clinical diagnoses of dementia made according to Diagnostic and Statistical Manual of Mental Disorders (DSM) IIIR criteria.^22^ This standardisation allowed a comparison of estimates without the unknown bias introduced by recent shifts in clinical attitudes and practice. Missing data within an interview could prevent an algorithm diagnosis and for individuals with missing data, the same approach was taken for CFAS II as for CFAS I, which was a review of all available information by diagnostician (CB), applying DSM-IIIR criteria. Many of these individuals had severe cognitive impairment and were not able to respond to the interview questions.

### Statistical analysis

We estimated prevalence using inverse probability weighting methods (in Stata, version 12) that adjusted for non-response (CFAS I and CFAS II) and study design selection (CFAS I). We calculated response by birth cohort and sex (CFAS I), and birth cohort, sex, care setting, and deprivation status of postcode (CFAS II), which were all factors that were known for the complete population, including those who did not take part. The effect of response adjustment on CFAS I was slight, so we did not use a more complex adjustment. In CFAS II, individuals who needed informant (proxy) interview because of known dementia (or other severe frailty) were weighted separately to account for this additional information.

We standardised prevalence estimates by the UK age and sex distribution at the time of the first interviews (1991 for CFAS I, 2011 for CFAS II) using 5 year age bands; for CFAS I estimates were then also standardised to the 2011 distribution. Prevalence estimates from CFAS II were applied using the age and sex distribution to generate population levels of dementia for the UK. We tested area variation for both time periods, also taking into account deprivation, using postcode-level Townsend deprivation scores.^24^ Prevalence estimates by deprivation tertile, age, and sex were applied to the age and sex distribution of England's upper tier local authorities (eg, counties or cities) to provide standardised estimates. Maps were drawn using quintiles of prevalence estimates.

We did a range of sensitivity analyses with inverse probability weighting methods and also using full likelihood Bayesian modelling (using WinBUGS software, version 1.4.3). Initially the inverse probability rates were checked against a similar model with missing interviews modelled using the same factors that generated the weight. We made further extensions to assume all individuals who did not respond had either a 50% or 100% higher occurrence of dementia (informative missing); we also modelled different types of non-response, with active refusal having lower dementia occurrence (20% lower), and increases in those who refused because they were too ill (100% higher) or who had a passive or proxy refusal (50% higher). These values were decided a priori on the basis of previous investigations of missing data.^26^ Further details of the methods are provided in the appendix.

### Role of the funding source

The funders are represented on the CFAS Management Committee and the Biological Resource Advisory Committee but they had no role in study design, data collection, data analysis, data interpretation, or writing of the report. The first author had full access to all the data in the study and the corresponding author had final responsibility for the decision to submit for publication.

## Results

In CFAS I, 7635 people aged 65 years and older were interviewed (9602 approached, 80% response) in Cambridgeshire, Newcastle, and Nottingham, with 1457 being diagnostically assessed. In the same geographical areas, 7796 were interviewed for CFAS II (14 242 approached, 242 with limited frailty information, 56% response). Thus, response was lower for CFAS II than for CFAS I. A comparison of the reasons for refusal suggests non-response across the cognitive spectrum, with reasons ranging from carers deeming an individual to be too frail to a lack of time. We identified some variation by area (appendix). Table 1 shows the numbers included within the analyses. As expected,^27^ there was a reduction in the proportion of the population to be approached residing in care settings (residential and nursing homes; 5% for CFAS I *vs* 3% for CFAS II; details in appendix).

**Table 1:** Demographic characteristics of individuals participating in screen and assessment (CFAS I) and interview (CFAS II)

	**CFAS I**		CFAS II*
	Screening*	Assessment†	
**Sex**			
Men	3045 (39%)	531 (38%)	3550 (44%)
Women	4590 (61%)	926 (62%)	4246 (56%)
**Age group**			
65–69 years	1981 (25%)	310 (23%)	1939 (23%)
70–74 years	1776 (23%)	320 (22%)	1874 (23%)
75–79 years	1725 (22%)	263 (23%)	1623 (21%)
80–84 years	1308 (18%)	291 (20%)	1289 (17%)
85–89 years	615 (9%)	186 (9%)	769 (11%)
≥90 years	230 (4%)	87 (3%)	302 (6%)
**Location**			
Cambridgeshire	2601 (34%)	465 (37%)	2558 (30%)
Newcastle	2522 (33%)	499 (31%)	2616 (34%)
Nottingham	2512 (33%)	493 (32%)	2622 (35%)
**Residential status**‡			
Community	7281 (95%)	1269 (95%)	7599 (97%)
Care homes	347 (5%)	183 (5%)	197 (3%)

Data are n (%). CFAS=Cognitive Function and Ageing Study.

* Percentages back-weighted for non-response.

† Percentages back-weighted for sampling design and non-response.

‡ Residential status missing for seven individuals for CFAS I (of whom five were also assessed).

**Figure 1:**
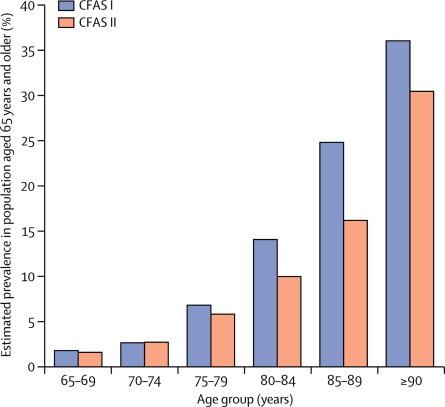
CFAS I and CFAS II age-specific dementia prevalence CFAS=Cognitive Function and Ageing Study.

Comparison of standardised prevalence across time showed a substantial decrease in prevalence of dementia (odds ratio [OR] CFAS II *vs* CFAS I was 0·7, 95% CI 0·6–0·9, p=0·003, adjusted for age, sex, area, and deprivation status; table 2, figure 1). Women had a consistently higher prevalence of dementia than men in all settings (table 3). The overall decrease was driven by non-care settings and was not noted within people in care settings, where prevalence increased (OR 1·7, 95% CI 1·0–2·9, p=0·05; table 3). In CFAS I, the number of individuals with dementia in care settings represented 34% of all dementia cases (29% men, 36% women). In CFAS II, despite higher dementia prevalence in care settings, the proportion of the population who had dementia and who lived in care had reduced slightly to 29% overall (17% men, 35% women). This apparent paradox is because of the reduction in the proportion of the older population who now live in care settings.

**Table 2:** Number with known dementia status and dementia prevalence

	**CFAS I**		CFAS II*	
	N	% (95% CI)	N	% (95% CI)
**Men**				
65–69 years	145	1·7% (0·8–3·2)	968	1·2% (0·6–2·3)
70–74 years	146	2·2% (1·2–4·0)	903	3·0% (2·0–4·4)
75–79 years	107	5·7% (3·4–9·2)	756	5·2% (3·8–7·0)
80–84 years	74	14·6% (8·6–23·7)	540	10·6% (8·2–13·7)
85–89 years	48	19·8% (10·3–34·6)	273	12·8% (9·0–18·0)
≥90 years	11	68·2% (34·8–89·6)	85	17·1% (10·6–26·4)
**Women**				
65–69 years	165	2·0% (1·0–3·8)	971	1·8% (0·9–3·6)
70–74 years	174	2·9% (1·7–5·0)	971	2·5% (1·6–3·9)
75–79 years	156	7·4% (3·7–14·6)	866	6·2% (4·5–8·4)
80–84 years	217	13·9% (9·7–19·4)	736	9·5% (7·3–12·3)
85–89 years	138	26·5% (18·1–37·1)	434	18·1% (14·5–22·2)
≥90 years	76	32·3% (21·7–45·0)	217	35·0% (28·4–42·3)
**By sex, standardised to 2011 UK age structure**				
Men	..	7·4% (5·7–9·1)	..	4·9% (4·2–5·7)
Women	..	9·4% (7·6–11·2)	..	7·7% (6·8–8·5)
Total	..	8·3% (7·0–9·6)	..	6·5% (5·9–7·0)
**By area, standardised to 2011 UK age structure**				
Cambridgeshire	..	6·7% (5·0–8·4)	..	6·1% (5·1–7·1)
Newcastle	..	8·3% (6·3–10·2)	..	7·2% (6·2–8·3)
Nottingham	..	10·2% (7·4–13·0)	..	6·0% (5·0–6·9)

* 76 individuals in CFAS II had unknown dementia status.

**Table 3:** Number with known dementia status and dementia prevalence by age, sex, and residential status

		**CFAS I**		**CFAS II**	
		N*	% (95% CI)	N	% (95% CI)
**Men**					
Community					
	65–74 years	274	0·9% (0·5–1·8)	1859	1·8% (1·3–2·7)
	75–84 years	166	8·1% (5·6–11·8)	1273	6·4% (5·2–8·1)
	≥85 years	40	15·0% (7·1–29·0)	339	11·9% (8·6–16·3)
	Overall	..	4·4% (3·3–5·8)	..	4·7% (4·1–5·7)
	Standardised	..	6·1% (4·1–8·1)	..	4·2% (3·5–4·9)
Care homes					
	65–74 years	17	59·7% (33·8–81·1)	12	34·0% (7·3–56·5)
	75–84 years	14	19·6% (5·6–50·2)	23	72·0% (44·6–87·0)
	≥85 years	18	83·9% (52·5–96·1)	19	55·6% (31·5–77·4)
	Overall	..	51·3% (31·0–71·3)	..	57·2% (39·8–68·7)
	Standardised	..	55·8% (45·6–65·9)	..	46·2% (27·7–58·8)
**Women**					
	Community				
	65–74 years	329	2·1% (1·3–3·3)	1935	1·6% (1·2–2·6)
	75–84 years	318	7·9% (5·1–12·1)	1563	5·9% (4·9–7·8)
	≥85 years	142	19·0% (12·1–28·6)	560	17·0% (14·2–21·9)
	Overall	..	7·1% (5·3–9·4)	..	6·0% (5·4–7·3)
	Standardised	..	6·7% (4·9–8·5)	..	5·5% (4·7–6·3)
Care homes					
	65–74 years	9	41·5% (15·3–73·6)	7	90·2% (50·3–98·8)
	75–84 years	55	62·7% (46·4–76·5)	39	76·0% (59·3–87·2)
	≥85	70	56·4% (38·7–72·6)	91	71·1% (60·0–80·1)
	Overall	..	58·0% (45·9–69·2)	..	73·4% (64·6–80·7)
	Standardised	..	55·9% (36·9–74·8)	..	75·2% (58·6–91·7)
**Total**					
Care homes					
	65–74 years	..	52·9% (33·3–71·6)	..	60·3% (35·9–80·5)
	75–84 years	..	51·4% (35·9–66·6)	..	74·6% (61·3–84·5)
	≥85 years	..	60·8% (44·3–75·1)	..	68·8% (58·7–77·5)
	Overall	..	56·4% (45·9–66·3)	..	69·6% (62·0–76·2)
	Standardised	..	59·7% (48·9–70·4)	..	64·6% (51·7–77·6)

* Residential status missing for five individuals in CFAS I.

**Figure 2:**
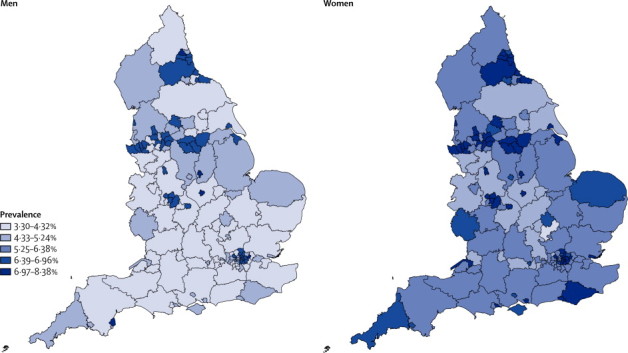
Estimated dementia prevalence in the UK in 2011, by sex and local authority

The analysis shows marginal, non-significant, differences across the three areas. Any difference disappears once area deprivation is taken into account (data not shown). However, figure 2 shows that predicted prevalence, adjusted for age structures and deprivation, varies widely across the country and between men and women.

Using the age and sex specific prevalence estimates from CFAS I in 1991, in the UK 664 000 individuals were expected to have dementia at that time. Taking into account only the effects of population ageing, this number would now be expected to be 884 000. However, CFAS II estimates that the number of people with dementia in the UK in 2011 was 670 000, 214 000 fewer than population ageing alone would have predicted, a reduction of 24%.

As detailed in the Methods and appendix, we did various sensitivity analyses. Assuming that all individuals living in care settings who were selected to take part, but did not**,** had dementia did not increase the estimates (table 4). The full likelihood Bayesian model gave very similar estimates with the assumption of missing at random and the inverse probability weighting method. Scenarios for informative missingness models were created, which tested how extreme assumptions would need to be to approach the earlier estimates. These scenarios did increase the estimates (table 4) but no scenarios, decided a priori and improbably severe, increased the prevalence to that estimated 20 years ago. These scenarios were deemed improbably severe because interviewers made efforts to seek a direct refusal and when there was gatekeeping, the nature of the frailty of the respondent was apparent. Thus, although we have no direct information for these individuals, the refusal codes allow for testing of our assumptions (further information provided in appendix).

**Table 4:** Sensitivity analyses for effect of sample non-response on estimate of dementia prevalence in 2011

	**Total**		**Men**		**Women**		**Cambridgeshire**	**Newcastle**	**Nottingham**
	Prevalence	Number	Prevalence	Number	Prevalence	Number			
Main analysis	6·5%	670 000	4·9%	227 000	7·7%	443 000	6·1%	7·2%	6·0%
Dynamic population	6·6%	685 000	5·0%	230 000	7·9%	455 000	6·3%	7·3%	6·1%
All in care not seen have dementia	5·9%	598 000	4·8%	222 000	6·8%	396 000	5·4%	6·8%	5·6%
Simple MAR*	6·0%	625 000	4·8%	221 500	7·0%	404 000	5·3%	7·1%	5·5%
Type of refusal MAR*	6·0%	625 000	4·8%	221 000	7·0%	403 500	5·3%	7·1%	5·5%
All refusals at 50% increased risk of dementia*	7·7%	797 000	6·1%	279 000	8·8%	516 500	6·7%	9·0%	7·2%
All refusals at 100% increased risk of dementia*	8·5%	882 000	6·7%	309 000	9·9%	572 000	7·4%	10·0%	8·0%
Ill health at 100% increased risk**,** passive or proxy refusals at 50% increased risk, active refusals at 20% decreased risk*	6·9%	717 000	5·5%	253 000	8·0%	464 000	6·1%	8·1%	6·4%

Descriptions of scenarios are provided in Methods and appendix. MAR=missing at random.

* Bayesian model.

## Discussion

This study provides compelling evidence of a reduction in the prevalence of dementia in the older population over two decades. On the basis of the age and sex specific prevalence estimates from CFAS I, 664 000 individuals were estimated to have dementia in 1991. Taking into account only the effects of population ageing, this number would now be expected to be 884 000. However, the results of CFAS II suggest that the number of people with dementia in 2011 was 670 000, which is 214 000 fewer than population ageing alone would have predicted—a reduction of 24%. However, the prevalence of dementia within care settings has increased from 56% to 70%. Prevalence varies according to deprivation indices and applying this information to English localities shows substantial variation in expected prevalence of dementia (panel).

Panel **Research in context**

**Systematic review**

We searched PubMed (up to July 11, 2013) with the search terms “dementia” or “Alzheimer's disease” and “cohort change” or “time” or “trends”. No studies were excluded because of language or year, but only studies relating to time changes seen in the recent past were selected for inclusion in this report. Studies were generally single site, or of specific age groups, or using different diagnostic criteria over time. No UK results were identified.

**Interpretation**

Results of a few studies^6^, ^7^, ^8^, ^15^, ^16^, ^28^ have suggested that in higher income countries, the prevalence and inferred or measured incidence of dementia and severe cognitive impairment might have decreased. Our findings support these results and also show that there is measurable variation in the prevalence of dementia within England. The scale of the reduction that we identified is substantial and is in line with major reductions in risk factors in higher income countries, which have been modified by societal changes such as improvements in education, and prevention and treatment strategies in recent decades.^29^

Although many factors could have increased dementia prevalence at specific ages (such as those associated with diabetes, survival after stroke, and vascular incidents), other factors, which could decrease prevalence, such as improved prevention of vascular morbidity and higher levels of education, seem to have had a greater effect. Further work will be done to attempt to quantify some of these changes, but this analysis is beyond the scope of the present paper. CFAS I and CFAS II were designed to investigate generational change and provide contemporaneous estimates. In the UK, substantial evidence exsits for inequality in health, and the present findings suggest that some areas will have benefited more than others from reduction in risk. Figure 2 shows that across England this variation, applied to local age structures, leads to differences in expected prevalence by geography. The numbers with manifest dementia in the population for planning and policy analysis, according to our study methods, will need to be revised downwards. Prevalence in care settings has increased, but most individuals with dementia still live in the community.

This evidence is based on repeated prevalence studies in randomly sampled older people living in the community or in care settings in three geographical areas of England. Dementia by age and sex shows the same patterns, but with lower point estimates. Prevalence has increased in care settings, which now contain a smaller proportion of the older population. Slight, non-significant, geographical variation was noted in both CFAS I and CFAS II and is explained by strong deprivation effects. The key features of CFAS II are that it was designed to examine cross-generational changes in dementia prevalence and related features, was done in several areas, was powered to detect changes, and had consistent methods and diagnostic procedures.

The studies had several limitations. There have been substantial changes in perceptions and attitudes to population-based research, with many more barriers for CFAS II, some of which are related to increased sensitivity about data protection. The accuracy of population registers is problematic in all areas. Reasons for non-response were not particularly different between these studies, but some reasons became more prominent, including both refusal by others for frail individuals, and refusal by very active individuals. There might have been further reasons that we could not observe or note. This reduction in response is a common theme in attempted population-based studies and has required detailed sensitivity analyses to assess its potential effect.^25^ None of the sensitivity scenarios (table 4) resulted in higher estimates of prevalence than those recorded 20 years ago, despite high prevalence of dementia being assumed in non-responders. The scenarios used, although based on knowledge of longitudinal attrition and drawing on as much information from the interviewers' responses as possible, cannot replace high response rates.^26^ Other scenarios have been explored to investigate potentially higher prevalence of dementia within those who did not respond to the interview; however, to reach the prevalence of dementia estimated in CFAS I, for those aged 80 years or older, at least 50% of non-responders would have had to have had dementia, which is unlikely in view of the study design with face-to-face contact whenever possible and for most refusals.

Although the key methods were identical, CFAS I was a two-stage study whereas CFAS II was one-stage, a decision made on the basis of experience of both attrition between phases and complexity of analysis in longitudinal studies. Most estimates in the scientific literature from two-stage studies do not fully take design features into account, leading to overoptimistic CIs, which do not account for the uncertainty introduced in multistage processes.^30^ Our CFAS I analyses do take these factors into account. CFAS II was designed to avoid this complexity while retaining the ability to compare with CFAS I.

The algorithmic diagnostic method was designed and introduced to provide consistency and reasonable validity compared with clinical classifications, but became superseded by clinical consensus methods.^31^ Large-scale epidemiological studies are now revisiting the algorithmic approach after many years of onerous multidisciplinary clinical consensus conferences.^32^ The changing nature of investigations without standardised cutoffs or age norms, the absence of clear operationalisation of changing criteria, the rise of the mild cognitive impairment clinical classification and, most recently, the discussion surrounding DSM-5 create a very unstable environment to sustain constant consensus approaches.^22^ The effects of these boundary and investigational changes are unknown, and will affect who and how much of the population is labelled with a diagnosis.^33^, ^34^ These variations could act in both directions and could lead either to a reduction in the number of people given the full dementia diagnosis (with some being classified into the mild cognitive impairment categories), or an increase (as a result of investigations showing abnormalities, which in fact might be common in non-demented older people, reinforcing diagnosis). So, although the algorithmic approach can now be deemed either outdated or forward-thinking,^32^ it has been held constant and is the only diagnostic method that can avoid the potential, unknown, biases noted previously, allowing for robust comparisons. The absolute numbers of people with dementia at any time point will be indicative of the diagnostic fashions of that era, a situation which complicates epidemiological comparisons.

Our results suggest that the original intention of MRC CFAS, which was to provide a dedicated surveillance method to examine changes in dementia and related factors over time using constant methods and including the ability to incorporate recent diagnostic advances, should be actively pursued.^2^ Whether or not the gains that we have identified for the present older population will be borne out in later generations will probably depend on whether further improvements in primary prevention and effective health care for disorders that increase the risk of dementia can be achieved, including addressing inequalities.^35^, ^36^

## References

[1] Wimo A, Prince M World Alzheimer report 2010. The global economic impact of dementia. http://www.alz.co.uk/research/files/WorldAlzheimerReport2010.pdf.

[2] Prince M, Bryce R, Albanese E, Wimo A, Ribeiro W, Ferri CP (2013). The global prevalence of dementia: a systematic review and metaanalysis. Alzheimers Dement.

[3] House of Lords Committee on Public Service and Demographic Change (2013). Ready for ageing?. http://www.parliament.uk/business/committees/committees-a-z/lords-select/public-services-committee/report-ready-for-ageing/.

[4] Ferri CP, Prince M, Brayne C, for Alzheimer's Disease International (2005). Global prevalence of dementia: a Delphi consensus study. Lancet.

[5] Knapp M, Prince M Dementia UK 2007. http://www.alzheimers.org.uk/site/scripts/download_info.php?fileID=2.

[6] Schrijvers EM, Verhaaren BF, Koudstaal PJ, Hofman A, Ikram MA, Breteler MM (2012). Is dementia incidence declining? Trends in dementia incidence since 1990 in the Rotterdam Study. Neurology.

[7] Qiu C, von Strauss E, Bäckman L, Winblad B, Fratiglioni L (2013). Twenty-year changes in dementia occurrence suggest decreasing incidence in central Stockholm, Sweden. Neurology.

[8] Lobo A, Saz P, Marcos G, the ZARADEMP Workgroup (2007). Prevalence of dementia in a southern European population in two different time periods: the ZARADEMP project. Acta Psychiatr Scand.

[9] MRC CFAS (1998). Cognitive function and dementia in six areas of England and Wales: the distribution of MMSE and prevalence of GMS organicity level in the MRC CFA Study. Psychol Med.

[10] Saunders PA, Copeland JR, Dewey ME (1993). The prevalence of dementia, depression and neurosis in later life: the Liverpool MRC-ALPHA Study. Int J Epidemiol.

[11] National End of Life Care Intelligence Network (2010). Deaths from Alzheimer's disease, dementia and senility in England. http://www.endoflifecare-intelligence.org.uk/resources/publications/deaths_from_alzheimers.

[12] Marmot M Fair society, health lives. The Marmot Review. Strategic review of health inequalities in England post 2010. http://www.instituteofhealthequity.org/projects/fair-society-healthy-lives-the-marmot-review.

[13] Jones DS, Greene JA (2012). The contributions of prevention and treatment to the decline in cardiovascular mortality: lessons from a forty-year debate. Health Aff.

[14] Meng X, D'Arcy C (2012). Education and dementia in the context of the cognitive reserve hypothesis: a systematic review with meta-analyses and qualitative analyses. PLoS One.

[15] Rocca WA, Petersen RC, Knopman DS (2011). Trends in the incidence and prevalence of Alzheimer's disease, dementia, and cognitive impairment in the United States. Alzheimers Dement.

[16] Langa KM, Larson EB, Karlawish JH (2008). Trends in the prevalence and mortality of cognitive impairment in the United States: is there evidence of a compression of cognitive morbidity?. Alzheimers Dement.

[17] Christensen K, Thinggaard M, Oksuzyan A (2013). Physical and cognitive functioning of people older than 90 years: a comparison of two Danish cohorts born 10 years apart. Lancet.

[18] Sekita A, Ninomiya T, Tanizaki Y (2010). Trends in prevalence of Alzheimer's disease and vascular dementia in a Japanese community: the Hisayama Study. Acta Psychiatr Scand.

[19] Launer LJ, Andersen K, Dewey ME (1999). Rates and risk factors for dementia and Alzheimer's disease: results from EURODEM pooled analyses. EURODEM incidence research group and work groups. European studies of dementia. Neurology.

[20] Matthews F, Brayne C, MRC CFAS investigators (2005). The incidence of dementia in England and Wales: findings from the five identical sites of the MRC CFA study. PLoS Med.

[21] George DR, Whitehouse PJ, Ballenger J (2011). The evolving classification of dementia: placing the DSM-V in a meaningful historical and cultural context and pondering the future of “Alzheimer's”. Cult Med Psychiatry.

[22] Copeland JR, Dewey ME, Griffiths-Jones HM (1986). A computerized psychiatric diagnostic system and case nomenclature for elderly subjects: GMS and AGECAT. Psychol Med.

[23] Kay DW, Dewey ME, McKeith IG (1998). Do experienced diagnosticians agree about the diagnosis of dementia from survey data? The effects of informants' reports and interviewers' vignettes. Int J Geriatr Psychiatry.

[24] Townsend P, Phillimore P, Beattie A (1988). Health and deprivation: inequality and the north.

[25] Galea S, Tracey M (2007). Participation rates in epidemiologic studies. Ann Epidem.

[26] Chatfield MD, Brayne CE, Matthews FE (2005). A systematic literature review of attrition between waves in longitudinal studies in the elderly shows a consistent pattern of dropout between differing studies. J Clin Epidemiol.

[27] Banks L, Haynes P, Balloch S, Hill M Changes in communal provision for adult social care: 1991–2001. http://www.jrf.org.uk/publications/changes-communal-provision-adult-social-care-1991-2001.

[28] Manton KC, Gu XL, Ukraintseva SV (2005). Declining prevalence of dementia in the U.S. elderly population. Adv Gerontol.

[29] Capewell S, O'Flaherty M (2011). Rapid mortality falls after risk-factor changes in populations. Lancet.

[30] Dunn G, Pickles A, Tansella M, Vazquez-Barquero JL (1999). Two-phase epidemiological surveys in psychiatric research. Br J Psychiatry.

[31] Brayne C, Stephan BC, Matthews FE (2011). A European perspective on population studies of dementia. Alzheimers Dement.

[32] Weir DR, Wallace RB, Langa KM (2011). Reducing case ascertainment costs in U.S. population studies of Alzheimer's disease, dementia, and cognitive impairment–part 1. Alzheimers Dement.

[33] Erkinjuntti T, Ostbye T, Steenhuis R, Hachinski V (1997). The effect of different diagnostic criteria on the prevalence of dementia. N Engl J Med.

[34] Wu YT, Lee HY, Norton S (2013). Prevalence studies of dementia in mainland china, Hong Kong and Taiwan: a systematic review and meta-analysis. PLoS One.

[35] Murray CJ, Richards MA, Newton JN (2013). UK health performance: findings of the Global Burden of Disease Study 2010. Lancet.

[36] Jagger C, Matthews R, Lindesay J, Robinson T, Croft P, Brayne C (2009). The effect of dementia trends and treatments on longevity and disability: a simulation model based on the MRC Cognitive Function and Ageing Study (MRC CFAS). Age Ageing.

