# Supplementary material for: A two-decade comparison of prevalence of dementia in individuals aged 65 years and older from three geographical areas of England: results of the Cognitive Function and Ageing Study I and II
Source: Lancet. 2013 Oct 26;382(9902):1405–12. doi: 10.1016/S0140-6736(13)61570-6 (PMC3906607; doi:10.1016/S0140-6736(13)61570-6)
Supplement: Supplementary appendix [file mmc1.pdf]

THE LANCET

Supplementary appendix

This appendix formed part of the original submission and has been peer reviewed. We post it as supplied by the authors.

Supplement to: Matthews FE, Arthur A, Barnes LE, et al, on behalf the Medical Research Council Cognitive Function and Ageing Collaboration. A two-decade comparison of prevalence of dementia in individuals aged 65 years and older from three geographical areas of England: results of the Cognitive Function and Ageing Study I and II. *Lancet* 2013; published online July 16. http://dx.doi.org/10.1016/S0140-6736(13)61570-6.

Supplementary Material

CFAS I

Full details of MRC Cognitive Function and Ageing Study have already been published.[1-3] Information presented here is based on the three geographical areas in which a prevalence study was repeated.

Three of the original six areas' data were selected for CFAS I (Cambridgeshire, Newcastle, Nottingham) to enable direct comparison with CFAS II. 11,401 individuals were identified from population registers (Supplementary Table 1a). There was an overall 80% response at approach to interview, with a further response rate of 74% to the assessment interview (with known baseline characteristics).

	Cambridgeshire		Newcastle		Nottingham		Total	
**Total population**	9954		45356		42952		98262	
**Population sampled**	3601		3700		4100		11401	
**Population errors**	135		285		111		531	
**Ineligible/not needed**	0		76		650		10870	
**Eligible for approach**	3466		3339		3339		10144	
**Not available for approach***	190		279		165		542	
**Approached**	3276		2522		3174		9510	
**Active refusal+**	641		484		521		1646	
**Proxy/passive refusal+**	34		23		108		165	
**Too ill+**	0		31		33		64	
**Interviewed (or informant needed)**	2601	(79%)	2522	(82%)	2512	(79%)	7635	(80%)
**Respondent**	2545		2485		2478		7508	
**Informant only**	56		37		34		127	

* Included as passive refusal (if moved) or too ill (if dead) in analysis. + Information not routinely collected in CFAS I.

Supplementary Table 1a: Sample numbers and individuals included (CFAS I)

CFAS II

Sampling

Three areas are included in the study: the cities (and surrounds) of Newcastle upon Tyne and Nottingham, and Cambridgeshire (consisting of the rural area of East Cambridgeshire and Fenland centred on Ely and surrounding villages, excluding Cambridge). The sampling frame was the same as that used in the original CFAS enumeration. The sampling was undertaken twice to ensure the population base was as up to date for each year of interviewing. A population of 2,500 was the target in each area. Individuals born before 1946/1947 (depending on sample year) from each area was drawn from general practice records covering the same geographical base (CFAS has kept up with changes in practices). A stratified sample based on the age groups 65-74 and 75 years and over was used with 50% of the sample in each age group. The ascertainment of each sample used over-sampling to cope with individuals with incorrect registration and ineligibility, GP refusals, and refusals by the individuals or their carers. In Cambridgeshire the original study used different geographical areas for Year 1 and Year 2 and this was repeated. In Newcastle and Nottingham the same geographical area was used in both Year 1 and Year 2 of the sampling. A total of 4,000 individuals' names and addresses were sampled for each year in each area. Any individuals previously included in CFAS were available for the new cohort study as this is a complete re-enumeration of the population. Similarly there were approximately 73 individuals (3% of the Newcastle sample) that were also part of the ongoing 85+ study.[4] Interviewing methods have core similarities and in the case of such duplication, with appropriate consent, information already collected was used to minimise interview burden on individuals. The baseline interviews started in October 2008 and were all completed by September 2011. After two years, the respondents were approached to repeat the interviews.

Response

The population within the areas of Cambridgeshire and Nottingham had increased substantially over the last 20 years, though there has been a slight fall in the numbers of the older population living in the Newcastle area. Population enumeration errors are still detectable, though have reduced. Full information on response will be published separately, but of those approached 56% responded to the interview (Supplementary Table 1b).

	Cambridgeshire		Newcastle		Nottingham		Total	
**Total population**	11353		36931		59682		107966	
**Population sampled**	7220		7988		6679		21887	
**Population errors**	41		305		198		544	
**Ineligible/not needed**	2415		2227		880		5522	
**Eligible for approach**	4764		5456		5601		15821	
**Not available for approach***	487		476		616		1579	
**Approached**	4277		4980		4985		14242	
**Active refusal+**	1210		1679		1754		4643	
**Proxy/passive refusal+**	105		140		151		396	
**Too ill+**	352		458		365		1175	
**Interviewed (or informant needed)**	2610	(61%)	2703	(54%)	2715	(54%)	8028	(56%)
**Respondent**	2532		2550		2568		7650	
**Informant only**	26		66		54		146	
**Informant refused (or not identified)**	52		87		93		232	

* Included as passive refusal (if moved) or too ill (if dead) in analysis.

Supplementary Table 1b: Sample numbers and individuals included (CFAS II)

Approach and consent

Individuals ascertained from the general practitioners received, after approval from the GP, a joint introductory letter from the GP/study team; a patient information sheet and a photograph of the interviewer who would be approaching them. After approximately 7 days this was followed by a visit to their current residence (own home or care home) to ask potential participants if they would be interested in taking part in the study and to arrange a convenient appointment to conduct the interview. The interviewer went through the patient information sheet again and answered any questions that the participant or their families may have had before asking the respondent to give written informed consent to take part in the study.

In the event a person was unable to give full informed consent, we sought to identify a suitable person to act as consultee, either next of kin or family friend/carer who was asked for consent if they considered it would have been the wishes of the participant as per Mental Capacity Act 2005. Consent was also sought for permission to access medical notes and flagging and tracing at the Health and Social Care Information Centre, formally Office of National Statistics (ONS), HearCheck screening consent and consent for collection of a saliva sample (Oragene DNA) were also requested.

Interviews

The respondent interview was the combined screen and assessment developed for CFAS with selected additional questions (www.cfas.ac.uk). This provides the Automated Geriatric Examination for Computer Assisted Taxonomy (AGECAT) study diagnostics algorithm within a single interview, drawing on respondent and observer ratings. In 20% an informant interview was also conducted for the refinement of study diagnosis and to provide essential proxy information where respondents are unable to answer questions. The interview was administered using assisted computer direct data entry and had the following sections:

- Demographic characteristics – marital status, education, social class, social economic group, residential status and intellectual activity.
- Lifestyle variables – smoking and alcohol history (enhanced baseline questions), brief measure of physical activity.
- Health status including self-perceived health, self-reported chronic diseases (including heart disease, angina, diabetes, stroke, Parkinson disease, epilepsy and meningitis), Rose angina questionnaire and medication history.

- Functional limitations, disability and extended activities of daily living and objective assessments of physical function.
- Cognitive function: Mini Mental State Examination (MMSE)[5] and extended items, verbal fluency, executive function Cambridge Cognitive Examination (CAMCOG)[6]; depression, dementia and anxiety from the Geriatric Mental State AGECAT[7]
- Social support, social capital, care needs and social networks, including receipt of informal care.
- Measures of hearing and visual impairment (as in CFAS I), plus a further hearing test by HearCheck Screener to estimate hearing loss.
- Sputum specimen for DNA acquisition.
- Individuals were asked for permission to flag for death notification and embarkations at the Office of National statistics and access to health and social care records (as in CFAS I)
- An informant interview (20% sample of all respondents was weighted toward cognitive and functional impairment). The two interviews together consist of the GMS and History and Aetiology Schedule that is used by AGECAT.
- Receipt of health services, social services, special housing and disability benefits –questions relevant to policy was included based on retrospective questions on use of services to respondents and informants.

The saliva sample was collected with consent to be stored for research purposes investigating ageing which would include genetic (DNA) tests.

Informants undertook an interview called the History and Aetiology Schedule (HAS) which combines the most important parts of all the interviews, together with expanding the information on the course of any change in mental state.

Care Homes

The samples were selected from GP population lists serving whole geographical areas and is not biased in relation to care home residence. However it is clear than individuals who live in care homes have an additional layer for consent and particular reasons why their refusal patterns may be different.

Therefore all individuals (whether participating or not) have been identified and sampling weights will be adjusted for living in care homes.

Individuals were identified as living in institutions based on a number of search strategies, each group was verified to ensure correct coding.

1. Care homes mentioned in interview
2. Multiple individuals from single postcodes
3. Names of premises that match care homes
4. Additional care homes using local knowledge

	Cambridgeshire	Newcastle	Nottingham
**Total eligible sample**	**4764**	**5456**	**5601**
Living in own home	4634	5253	5391
Living in care home	115 (2.4%)	198 (3.6%)	192 (3.4%)
Living in care 2011 Census	1.5%	3.0%	2.8%
Response in care home	29 (25%)	97 (49%)	71 (37%)

Supplementary Table 2: Enumeration and response from individuals living in care homes

Generating the weight

Characteristics of response for the informant interviews

There are two groups of informant individuals, those who we were informed were too demented to interview and all the others. These two groups are considered separately initially.

Informant interviews (HAS) for individuals suspected of having dementia

Individuals identified by the GP, care home, family, respondent, interviewer or programme as being not able to be interviewed because they are too demented were treated separately from those who were informant only for other frailty conditions. All individuals identified in this way who had an interview with an informant were found to be demented. Therefore the weight ensured that those individuals without an informant interview are also included as dementia cases within the analysis. This is included as a sensitivity analysis in the main text of the paper.

There were 265 individuals identified as too demented to be interviewed and therefore needing informant interviews, 91 were successfully undertaken.

	No HAS		HAS done		Odds Ratio (95% CI)	
**Sex**						
Male	45	(58%)	32	(42%)	1.0	
Female	129	(69%)	59	(31%)	0.6	(0.3-1.2)
**Birth Cohort**						
<1930	120	(65%)	64	(35%)	1.0	
1930-1939	46	(67%)	23	(33%)	1.2	(0.6-2.3)
1940 onwards	8	(67%)	4	(33%)	1.0	(0.2-4.4)
**Area**						
Cambridgeshire	39	(78%)	11	(22%)	1.0	
Newcastle	60	(56%)	47	(44%)	2.1	(0.9-5.1)
Nottingham	75	(69%)	33	(31%)	1.5	(0.6-3.6)
**Residential Care**						
No	157	(79%)	34	(18%)	1.0	
Yes	17	(26%)	57	(77%)	17.8	(8.7-36.4)
**Deprivation**						
Least deprived	43	(67%)	21	(33%)	1.0	
Middle tertile	63	(62%)	38	(38%)	0.7	(0.3-1.5)
Most deprived	68	(68%)	32	(32%)	0.4	(0.2-1.0)

Supplementary table 3: Response characteristics for individuals with identified dementia

For individuals with dementia who lived in care homes an informant only interview was more likely to be the source of information, than for individuals with dementia in the community. The age of the respondent and deprivation had little influence on the likelihood of response of the informant.

Weights generated from this analysis ranged from 1.1-10.0, the lowest weight was for male respondents in residential care in Newcastle. The highest weights were for the oldest female respondents from Cambridgeshire who were not living in a care setting.

Informant interviews for individuals with other frailty

Individuals identified by the GP, care home, family, respondent, interviewer or programme as being not able to be interviewed because they are too frail were also considered separately than the rest of the general population. Individuals identified in this way who had an interview were very likely to have dementia. Therefore the weight ensured that these individuals without an informant interview are also included as at higher risk of dementia within the analysis.

There were 113 individuals identified as too frail to be interviewed and therefore needing informant interviews.

	No HAS		HAS done		Odds Ratio (95% CI)	
**Sex**						
Male	12	(41%)	17	(59%)	1.0	
Female	46	(55%)	38	(45%)	1.1	(0.3-3.2)
**Birth Cohort**						
<1930	41	(54%)	35	(46%)	1.0	
1930-1939	12	(41%)	17	(59%)	1.3	(0.4-3.7)
1940 onwards	5	(63%)	3	(38%)	0.4	(0.1-2.9)
**Area**						
Cambridgeshire	13	(46%)	15	(54%)	1.0	
Newcastle	27	(59%)	19	(41%)	0.8	(0.2-2.5)
Nottingham	18	(46%)	21	(54%)	1.2	(0.4-4.0)
**Residential Care**						
No	11	(24%)	35	(76%)	1.0	
Yes	47	(70%)	20	(30%)	0.1	(0.1-0.3)
**Deprivation**						
Least deprived	15	(50%)	15	(50%)	1.0	
Middle tertile	22	(69%)	10	(31%)	0.5	(0.1-1.5)
Most deprived	21	(41%)	30	(59%)	1.2	(0.4-3.7)

Supplementary table 4: Response characteristics for individuals with frailty due to reasons other than dementia

Individuals with frailty other than dementia in care homes were much less likely to have an informant interview than such individuals who were in the community. There was little evidence of other measured factors being associated with lack of response.

Weights generated from this analysis ranged from 1.2-7.9, the lowest weights were for respondents in the community in Cambridgeshire or Nottingham. The highest weights were for women living in care settings.

Generation of weights for those not identified as needing an informant interview

The rest of the sample is considered together. Individuals who did not have an interview for any reason were considered as having similar risk factors for non-response. A sensitivity analysis allowing for different response mechanisms is shown presented in the main body of the paper.

There were 15,443 individuals who were eligible to receive a respondent interview. The birth cohorts within the population were split into five groups and deprivation into quintiles for weight generation, but for ease of reading the table these have been combined here to be consistent to before.

	No interview		Interview		Odds Ratio (95% CI)	
**Sex**						
Male	3363	(49%)	3501	(51%)	1.0	
Female	4430	(52%)	4149	(48%)	0.9	(0.9-1.0)
**Birth Cohort**						
<1930	2635	(56%)	2097	(44%)	1.0	
1930-1939	3281	(49%)	3427	(51%)	1.3	(1.2-1.4)
1940 onwards	1877	(47%)	2126	(53%)	1.4	(1.3-1.5)
**Area**						
Cambridgeshire	2154	(46%)	2532	(54%)	1.0	
Newcastle	2753	(52%)	2550	(48%)	0.8	(0.7-0.8)
Nottingham	2886	(53%)	2568	(47%)	0.8	(0.7-0.8)
**Residential Care**						
No	7549	(50%)	7530	(50%)	1.0	
Yes	244	(66%)	120	(33%)	0.6	(0.5-0.8)
**Deprivation**						
Least deprived	2288	(44%)	2961	(56%)	1.0	
Middle tertile	2570	(50%)	2544	(50%)	0.8	(0.7-0.8)
Most deprived	2935	(58%)	2145	(42%)	0.6	(0.5-0.6)

Supplementary table 5: Response characteristics for individuals with identified other frailty

Individuals in care homes were less likely to take part than those in the community, increasing deprivation and increasing age were also associated with increased refusal. There was little difference between men and women. After adjustment for these there was a small area effect.

Weights generated from this analysis ranged from 1.7-4.6, the lowest weights were for respondents living in the community from Cambridgeshire. The highest weights were for the oldest respondents living in Newcastle and Nottingham.

Further investigation of non-response is the focus of another paper.

Sample weight generation

Each individual who has data within the interview (either respondent or informant) is included with their response rate as calculated above. The weights are then standardised to range from 1, though this makes little difference to the results. The final sample weights range from 1.0 to 7.6 a histogram is shown below (Supplementary Figure 1). The weights are concentrated around the mean and median of 1.7, with 90% of the weights between 1.5-2.0. This indicates that the sample of respondents represents the population sample well (if all individuals had the same weight there would be no effect of sample weights in the analysis).

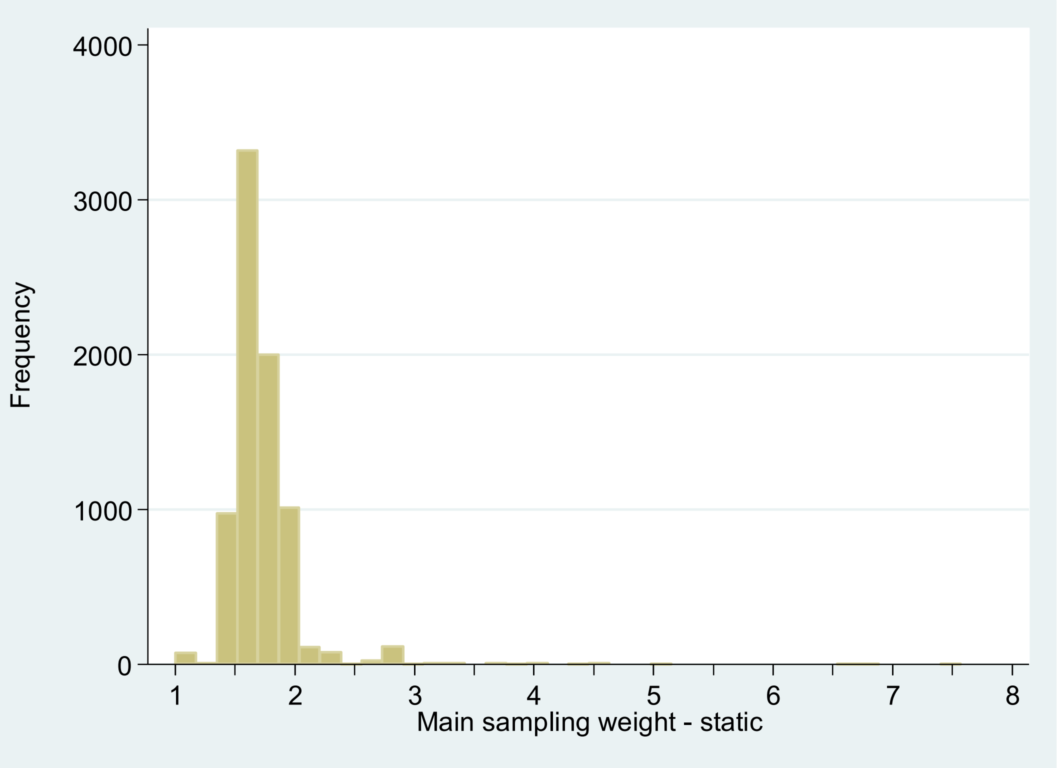

Supplementary Figure 1: Histogram of weights

Analytical methods

The majority of the analyses presented are based on weighted prevalence estimates from the groups illustrated. Information on the response weight is detailed above. Both studies then further weighted for the presence of known dementia diagnosis. The analysis used the svy commands within Stata 12. Logistic regression models were used to investigate the effect of area and deprivation after adjusting for age and sex differences. Cohort effect is investigated using a weighted logistic regression after adjustment for age, sex and area differences.

Sensitivity analyses were undertaken in two ways. Initially the robustness of the weighting strategy was investigated (data not shown, technical report available from the authors), further investigation of assuming dementia cases for individuals living in care homes who were not interviewed is also presented (Table 4). An analysis that assumes the population is that seen at approach (dynamic population) where all those who died or moved away between being sampled and being interviewed are not included within the denominator is shown. A reviewer raised the same concerns expressed by us within the paper about non-response bias. We have tried to undertake scenarios that are plausible based

on our response mechanism, and to that measureable from previous longitudinal studies. The question posed was what would the prevalence estimates need to be in the non-responders to produce the same estimates as seen in CFAS I. Prevalence and non response do both change with age. Our scenarios are based on a constant effect of non response across age but we have explored the amount of prevalence within age and gender groups that would be needed in the excess CFAS II non responders to reach or increase an age specific prevalence estimate as suggested by the reviewer. In the oldest men even if all non-responders were demented the prevalence would not be the same as in CFAS I, for all those aged over 80 the prevalence of dementia in the non-responders would have to exceed 50% for the prevalence in CFAS II to match that seen in CFAS I, the average standardised dementia prevalence in the excess non responders (i.e. those over and above the 20% non response in CFAS I) would need to be above 18%. Even at the youngest ages the prevalence would need to be 4.5%, much higher than 3% seen in CFAS I. Each age and sex specific group requires a different amount due to the nature of dementia and non-response, in men prevalence of dementia ranged from 1.5 times that seen (at youngest ages) to 14 times that seen, with an average of 4.8, in women the effects are more consistent across the age groups ranging from one to three times, with an average of double that seen.

Full likelihood Bayesian models of the prevalence estimate were also undertaken, using standard logistic regression with vague priors. Initially the model included a missing dementia diagnosis step with similar covariates to that used in the weight. Further extensions of this missing diagnosis can include informative missing processes where the prevalence of dementia is imputed with external information to reflect potential biases. All Bayesian models were run from three separate initial values, convergence was checked using standard techniques. All analyses had burn in of 5000 iterations with 10,000 iterations for the final analyses. The median and credible interval were calculated from the posterior distribution. The scenarios undertaken were that of a mixed risk where individuals who were ill had double the dementia risk, those with a proxy (or informant) refusal a 50% increase but those who were active refusals had a slightly decreased risk of dementia. In addition a very simple 100% increase in dementia prevalence for all individuals not seen, together with a lesser 50% increase. Results are presented in Table 4 and the main results shown in Supplementary Figure 2.

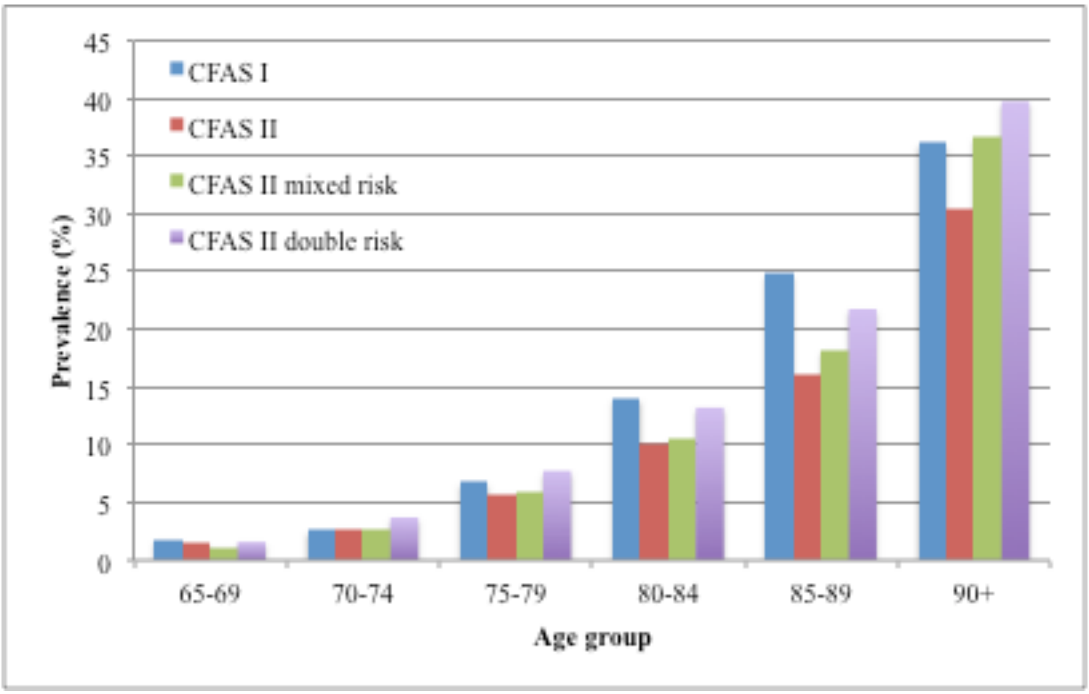

Supplementary Figure 2: Prevalence by age in CFAS I and CFAS II including informative missing scenarios

The dementia map is calculated from a logistic regression analysis of deprivation specific tertiles of age and sex prevalence. Each area is then mapped to one of the tertiles and the age specific rates imposed on that population (2011 Census). These results are standardised and then mapped using ArcGis.

References

1) Matthews FE, Brayne C, Investigators MRC CFAS. The Incidence of Dementia in England and Wales: Findings from the Five Identical Sites of the MRC CFA Study. *PLoS Med.* 2005; **2**: e193
2) MRC CFAS. Cognitive function and dementia in six areas of England and Wales: the distribution of MMSE and prevalence of GMS organicity level in the MRC CFA Study. *Psychological Medicine,* 1998; **28**:319-335
3) Brayne C; McCracken C; Matthews FE. Cohort Profile: The Medical Research Council Cognitive Function and Ageing Study (CFAS). *International Journal of Epidemiology* 2006; **35**: 1140-1145.
4) Collerton J, Davies K, Jagger C, Kingston A, Bond J, Eccles M, Robinson L, Martin-Ruiz C, Von Zglinicki T, James O, Kirkwood T**.** Health and disease in 85 year olds: Baseline findings from the Newcastle 85+ cohort study**.** *British Medical Journal*, 2009; **339**: b4904.
5) Folstein MF, Folstein SE, McHugh PR. "Mini-mental state". A practical method for grading the cognitive state of patients for the clinician. *J Psychiatr Res* 1975; **12**:189-98.
6) Huppert FA, Brayne C, Gill C, Paykel ES, Beardsall L. CAMCOG--a concise neuropsychological test to assist dementia diagnosis: socio-demographic determinants in an elderly population sample. *Br J Clin Psychol* 1995; **34**:529-41.
7) Copeland JR, Dewey ME, Griffiths-Jones HM. A computerized psychiatric diagnostic system and case nomenclature for elderly subjects: GMS and AGECAT. *Psychol Med*. 1986; **16**:89-99.
